# Integrated molecular and metabolomic assessment of copper hydroxide nanopesticide toxicity in zebrafish

**DOI:** 10.1007/s10695-026-01701-4

**Published:** 2026-05-22

**Authors:** Feyza Icoglu Aksakal, Cihan Gür, Turgay Şişman, Semih Özli, Onur Şenol, Özkan Aksakal

**Affiliations:** 1 Department of Agricultural Biotechnology, Faculty of Agriculture, Atatürk University, Erzurum, Turkey; 2 Department of Medical Laboratory Techniques, Vocational School of Health Services, Atatürk University, Erzurum, Turkey; 3 Department of Molecular Biology and Genetics, Faculty of Science, Atatürk University, Erzurum, Turkey; 4 Department of Analytical Chemistry, Faculty of Pharmacy, Atatürk University, Erzurum, Turkey

**Keywords:** Apoptosis, Copper homeostasis, Cu(OH)_2_ nanopesticide, Endoplasmic reticulum stress, Metabolomics

## Abstract

Copper hydroxide (Cu(OH)_2_) nanopesticides [Cu(OH)_2_ NP] are increasingly applied in agriculture, raising concerns about their potential risks to non-target aquatic organisms. In this study, we investigated the toxic effects of Cu(OH)_2_ NP exposure in zebrafish (*Danio rerio*) larvae and adults using an integrated gene expression and metabolomic approach. Zebrafish embryos were exposed to sublethal concentrations of Cu(OH)_2_ NP until 96 h post-fertilization, while adults were subjected to a 14-day sub-chronic exposure. Cu(OH)_2_ NP significantly altered the expression of genes involved in copper homeostasis and redox regulation (*cox16*, *atp7a*, *ccs*, and *gclm*), accompanied by activation of endoplasmic reticulum stress (*atf6*, *perk*, and *ire1*) and apoptosis-related pathways (*casp3*, *casp9*, *bax*, and *bcl2*). These responses were dose-dependent and more pronounced in zebrafish larvae and adult liver tissue. Metabolomic analysis revealed substantial metabolic reprogramming, particularly affecting energy metabolism, amino acid pathways, and glutathione metabolism. In adult zebrafish, significant changes in hepatosomatic index (HSI) indicated disrupted energy balance and hepatic stress. Integrated analysis suggests that Cu(OH)_2_ NP toxicity is associated with disruption of copper homeostasis and activation of cellular stress-response pathways. Overall, these findings improve our understanding of the molecular and metabolic responses of zebrafish to Cu(OH)_2_ NP exposure and highlight potential ecological risks of nano-enabled agrochemicals.

## Introduction

The extensive use of conventional pesticides in modern agriculture has played a critical role in maintaining crop productivity and global food security. However, the excessive and continuous application of these chemicals has resulted in serious environmental concerns, including contamination of aquatic ecosystems, adverse effects on human health, and biodiversity loss (Aksakal and Sisman 2020; Liu et al. 2014). In response to these challenges, nanotechnology-based approaches have been increasingly adopted in agricultural practices to enhance pesticide efficiency while potentially reducing application rates and environmental loading (Carley et al. 2020; Zhao et al. 2016). Nanopesticides, owing to their reduced particle size and altered surface properties, are designed to improve bioavailability, persistence, and target specificity compared to traditional formulations (Kah et al. 2018).

Among copper-based agrochemicals, copper hydroxide [Cu(OH)_2_] is widely applied as a fungicide and bactericide to control plant diseases. Although Cu(OH)_2_ has long been used in conventional formulations, its nano-enabled counterparts have recently gained prominence due to their enhanced adhesion to plant surfaces, controlled copper ion release, and prolonged efficacy (Zhao et al. 2016). Despite these advantages, increasing evidence suggests that Cu(OH)_2_ NP may exhibit distinct toxicological profiles compared to dissolved copper ions, raising concerns regarding their unintended impacts on non-target organisms, particularly in aquatic environments (Chen et al. 2023).

Following agricultural application, Cu(OH)_2_ NP can enter freshwater systems through runoff, leaching, and drainage, where they may persist and bioaccumulate. Previous studies have demonstrated that exposure to Cu(OH)_2_ NP adversely affects aquatic organisms, including fish and invertebrates, by disrupting development, metabolism, immune function, and behavior (Aksakal and Arslan 2020; Chen et al. 2023; Wang et al. 2021, 2024a, b). In zebrafish, Cu(OH)_2–_NP exposure has been shown to impair embryonic development, alter neurotransmitter pathways, disturb energy metabolism, and induce oxidative stress. Notably, several studies indicate that the biological responses elicited by Cu(OH)_2–_NP differ from those induced by copper ions alone, suggesting nano-specific mechanisms of toxicity (Aksakal and Sisman 2020; Chen et al. 2023; Gulmez et al. 2023; Wang et al. 2021, 2024a, b).

Copper is an essential trace element involved in mitochondrial respiration, antioxidant defense, and numerous enzymatic reactions. However, disruption of copper homeostasis can lead to cellular toxicity through oxidative stress, mitochondrial dysfunction, and proteostasis imbalance (Gui and Wang 2025). Recent advances have highlighted the role of copper dysregulation in triggering endoplasmic reticulum (ER) stress and mitochondrial-mediated apoptosis (Zhao et al. 2020a, b). In this context, alterations in copper transport, intracellular trafficking, and redox regulation may serve as key upstream events linking Cu(OH)_2–_NP exposure to downstream cellular damage.

The zebrafish (*Danio rerio*) is a well-established vertebrate model for evaluating the toxicological effects of environmental contaminants due to its genetic homology to mammals, rapid development, and sensitivity to chemical stressors (Aksakal and Ciltas 2019). Importantly, zebrafish embryos and adults allow for integrative assessment of molecular, metabolic, and tissue-specific responses to toxicants. While previous studies have characterized developmental, neurotoxic, and metabolic effects of Cu(OH)_2_ NP in zebrafish, the mechanistic links between copper homeostasis, metabolic reprogramming, ER stress, and apoptosis remain insufficiently understood.

Therefore, the present study aimed to elucidate the molecular and metabolic mechanisms underlying Cu(OH)_2_ NP toxicity in zebrafish by integrating gene expression analysis with metabolomic profiling. Specifically, we investigated (i) the effects of Cu(OH)_2_ NP exposure on genes associated with copper homeostasis, redox regulation, ER stress, and apoptosis in zebrafish larvae and adults and (ii) the metabolic alterations induced in 96 h post-fertilization larvae as well as in the liver and brain tissues of adult zebrafish; (iii) physiological condition indices such as condition factor (CF), body mass index (BMI), and hepatosomatic index (HSI) in adult zebrafish. By combining gene expression, physiologic, and metabolomic endpoints, this study provides mechanistic insight into how Cu(OH)_2_ NP disrupts cellular homeostasis and induces toxicity in aquatic vertebrates, contributing to improved ecological risk assessment of nano-enabled agrochemicals.

## Materials and methods

### Chemicals and reagents

Cu(OH)_2_ NP used in this study was a commercial formulation (Kocide 3000®) purchased from DuPont (Midland, MI, USA). According to the manufacturer and previous characterization studies (Aksakal and Arslan 2020; Aksakal and Sisman 2020; Gulmez et al. 2023), this formulation contains micron-sized copper particles together with nanoscale copper hydroxide sheets and nanoparticles. Stock suspensions were freshly prepared by dispersing Cu(OH)_2_ NP in embryo-rearing medium (E3) and homogenized by gentle stirring prior to each exposure. All other chemicals and reagents were of analytical grade.

### Zebrafish husbandry

Wild-type AB strain zebrafish (*Danio rerio*) were obtained from Atatürk University Faculty of Fisheries. Fish were maintained under standard laboratory conditions (14:10 h light/dark cycle, temperature 26–28 °C, pH 7.0–7.5, dissolved oxygen > 6.5 mg L⁻^1^). Adult zebrafish were fed twice daily with commercial fish food. All experimental procedures were conducted in accordance with the OECD guidelines and approved by the Institutional Animal Ethics Committee of Atatürk University (E-75296309–050.01.04–2200399152, No 269).

### Embryo collection

For embryo production, adult zebrafish were placed in breeding tanks at a ratio of one male to two females the evening before spawning. Fertilized eggs were collected the following morning within 1 h post-fertilization (hpf), rinsed twice with E3 medium, and examined under a stereomicroscope. Only normally developing embryos were selected for exposure experiments. Embryos at 1.5 hpf were used for developmental toxicity assays.

### Embryonic exposure to Cu(OH)_2_ NP

Embryonic toxicity assays were conducted following OECD Test Guideline No. 236 (Test No. 236: Fish Embryo Acute Toxicity (FET) Test 2025). Groups of 30 embryos were placed in 30-mL glass Petri dishes containing E3 medium (control) or Cu(OH)_2_ NP at sublethal concentrations (0.25, 0.50, and 1.00 mg L⁻^1^), selected based on previously reported LC₅₀ values (Aksakal and Sisman 2020). Exposures were carried out from 1.5 hpf to 96 hpf under semi-static conditions, with daily renewal of exposure solutions. Mortality and developmental progression were recorded every 24 h, and dead embryos were removed. Each treatment was performed in triplicate. At the end of exposure, larvae were washed with ultrapure water, anesthetized on ice, snap-frozen, and stored at − 80 °C for molecular and metabolomic analyses.

### Adult zebrafish exposure Cu(OH)_2_ NP

Sub-chronic toxicity tests in adult zebrafish were conducted according to OECD guideline No. 204 (OECD 2012). Approximately 5-month-old zebrafish (0.3–0.4 g) were acclimated for 2 weeks prior to exposure. Fish were randomly distributed into four groups (*n* = 15 per group): control (clean system water), 0.5, 1.0, and 2.0 mg L⁻^1^ Cu(OH)_2_ NP. Exposures were carried out for 14 days under semi-static conditions in glass aquaria (30 × 15 × 20 cm) containing 5 L of system water. Test solutions were renewed every 24 h, and water quality parameters were monitored throughout the experiment.

The 14-day exposure duration was selected in accordance with OECD Guideline No. 204 for sub-chronic toxicity testing in fish (OECD 2012). The decision to perform terminal sampling on Day 14 was strategically aimed at capturing the integrated molecular and metabolomic steady-state of the organism. While multi-point sampling can track acute fluctuations, a 14-day terminal analysis provides a comprehensive assessment of the cumulative physiological burden, allowing for the detection of sustained metabolic reprogramming and chronic cellular stress responses (e.g., ER stress and oxidative stress) that may not be fully established during the initial acute phase of exposure (Jian et al. 2024; Wang et al. 2021).

### Measurement of copper content

Internal copper accumulation in zebrafish larvae and adult tissues was quantified to evaluate the uptake of Cu(OH)_2_ NP–derived copper. At the end of the exposure period, pooled larvae (*n* = 30 per replicate) and dissected adult liver and brain tissues were collected, rinsed with ultrapure water to remove surface-bound residues, and dried at 60 °C to constant weight. Dried samples were digested with concentrated nitric acid (HNO_3_, 65%) under controlled heating conditions until complete mineralization was achieved. Following digestion, samples were diluted with ultrapure water to a final volume and filtered prior to analysis.

Copper concentrations were determined using inductively coupled plasma-mass spectrometry (ICP-MS, Agilent 7800 ICP-MS). Calibration was performed using certified copper standard solutions, and quality control samples were included to ensure analytical accuracy. Copper content was expressed as µg Cu per g dry weight for larvae and adult tissues.

### RNA isolation and gene expression analysis

Total RNA was isolated from pooled larvae (*n* = 30 per replicate) and from adult liver and brain tissues using a commercial RNA isolation kit according to the manufacturer’s protocol, as described by Aksakal and Sisman (2020). RNA concentration and purity were assessed spectrophotometrically. First-strand cDNA synthesis was performed using a cDNA synthesis kit. Quantitative real-time PCR (qRT-PCR) was conducted using SYBR Green chemistry on a StepOnePlus™ Real-Time PCR System (Applied Biosystems). Gene expression levels of copper metabolism–related genes (*cox16*, *atp7a*, *ccs*, *gclm*), endoplasmic reticulum stress markers (*atf6*, *perk*, *ire1*), and apoptosis-related genes (*caspase-3*, *caspase-9*, *bax*, *bcl-2*) were analyzed. β-actin was used as the reference gene (Table 1). Relative gene expression was calculated using the 2^−ΔΔCt^ method.

**Table 1:** Primer sequences used for quantitative real-time PCR analysis

Gene	Forward/reverse primer (5′−3′)	Size (bp)	Accession No
*Atf-6*	F: CTGTGGTGAAACCTCCACCT R: CATGGTGACCACAGGAGATG	200	NM_001110519
*Perk*	F: TGGGCTCTGAAGAGTTCGAT R: TGTGAGCCTTCTCCGTCTTT	193	XM_005156585.4
*Ire1*	F: TGACGTGGTGGAAGTTGGTA R: ACGGATCACATTGGGATGTT	199	XM_021471110.1
*Caspase-3*	F: CCGCTGTGCTTCATTAGTGTG R: TCCAGTTCTGTTCCTCGACAAG	180	NM_131877.3
*Caspase-9*	F: CAGCACAGCGTCTGATGAACTT R: TCCTCCAGCACACGATCAAGAT	153	NM_001007404.2
*Bax*	F: ACAGGGATGCTGAAGTGACC R: GAAAAGCGCCACAACTCTTC	236	NM_131562.2
*Bcl-2*	F: TCACTCGTTCAGACCCTCAT R: ACGCTTTCCACGCACAT	235	NM_001030253.2
*Cox16*	F: AGGATTTGGACGGATGGA R: TGTTCAGTGTTATGTGCC	–-	(Gui and Wang 2025)
*Atp7a*	F: GTGGTGGTTCTTCTGGTTG R: TGCCTCGGAGGTCTTACT	–-	(Gui and Wang 2025)
*Ccs*	F: AATCACCCGCTGTCCAAA R: TCACTCCATCGCAGGCAC	–-	(Gui and Wang 2025)
*Gclm*	F: GAGCAATGGAGCATCACG R:TCCTCACTGGGCGAAGACG	–-	(Gui and Wang 2025)
*β-actin*	F: TTCACCACCACAGCCGAAAGA R: TACCGCAAGATTCCATACCCA	223	NM_131031.2

### Metabolomic analysis

Untargeted metabolomic analysis was performed on 96 hpf larvae and on adult liver and brain tissues. For each group, 25 larvae or pooled tissue samples were homogenized in ice-cold extraction solvent (methanol). Homogenization was maintained in TissueLyser 2.0. Samples were incubated for 30 min for cell lysis at − 20 °C and sonicated for 10 min. Next, centrifugation was applied at 12,500 rpm for 10 min. Supernatant was gently collected from the residue and transferred to another microcentrifuge tube. All analytes were dried under vacuum and reconstituted via mobile phase (acetonitrile:water, 70:30, v/v). Metabolic profiling was conducted using a Q-TOF mass spectrometry system in positive ESI mode. Multivariate statistical analyses, including principal component analysis (PCA) and orthogonal partial least squares–discriminant analysis (OPLS-DA), were applied to identify exposure-related metabolic alterations with the aid of MATLAB 2021b. Pathway enrichment analysis was performed to determine significantly affected metabolic pathways via MetaboAnalyst 6.0.

### Biometric measurements

At the end of the exposure period, adult zebrafish were anesthetized (MS-222), and total body weight and length were measured. Condition factor (CF), body mass index (BMI), and hepatosomatic index (HSI) were calculated using the following formulas:

$$\begin{array}{l}CF=\mathrm{b}\mathrm{o}\mathrm{d}\mathrm{y}\, \mathrm{w}\mathrm{e}\mathrm{i}\mathrm{g}\mathrm{h}\mathrm{t} \left(g\right)\times 100/\mathrm{b}\mathrm{o}\mathrm{d}\mathrm{y} \,\mathrm{l}\mathrm{e}\mathrm{n}\mathrm{g}\mathrm{t}\mathrm{h} {\left(cm\right)}^{3}\\ BMI=\mathrm{b}\mathrm{o}\mathrm{d}\mathrm{y} \,\mathrm{w}\mathrm{e}\mathrm{i}\mathrm{g}\mathrm{h}\mathrm{t} \left(g\right)/100/\mathrm{b}\mathrm{o}\mathrm{d}\mathrm{y}\, \mathrm{l}\mathrm{e}\mathrm{n}\mathrm{g}\mathrm{t}\mathrm{h} {\left(cm\right)}^{2}\\ HSI=\mathrm{l}\mathrm{i}\mathrm{v}\mathrm{e}\mathrm{r}\, \mathrm{w}\mathrm{e}\mathrm{i}\mathrm{g}\mathrm{h}\mathrm{t} \left(g\right)\times 100/\mathrm{b}\mathrm{o}\mathrm{d}\mathrm{y} \,\mathrm{w}\mathrm{e}\mathrm{i}\mathrm{g}\mathrm{h}\mathrm{t} \left(g\right)\end{array}$$

Subsequently, fish were dissected, and liver and brain tissues were rapidly excised, rinsed with ultrapure water, frozen in liquid nitrogen, and stored at − 80 °C for further analyses.

### Statistical analysis

All data were expressed as mean ± standard deviation (SD). Statistical analyses were performed using SPSS 22.0 software. Data were tested for normality and homogeneity of variance prior to analysis. One-way analysis of variance (ANOVA) followed by appropriate post hoc tests (Duncan, Dunnett, or Dunnett’s T3) was used to determine statistically significant differences between groups. A *p*-value < 0.05 was considered statistically significant.

## Results and discussion

### Copper accumulation in zebrafish larvae and adult tissues following Cu(OH)_2_ NP exposure

**Fig. 1:**
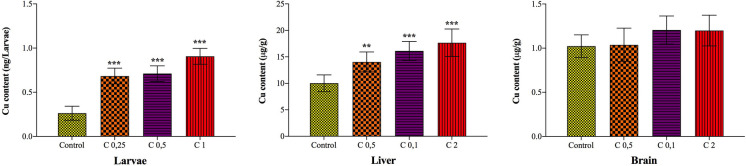
Copper accumulation in larvae and adult tissues (liver and brain) following exposure to Cu(OH)_2_ NP

Exposure to Cu(OH)_2_ NP resulted in a clear, concentration-dependent increase in internal copper levels in both zebrafish larvae and adults (Fig. 1). In larvae exposed until 96 hpf, whole-body copper content increased progressively with increasing exposure concentration, demonstrating that Cu(OH)_2_ NP–derived copper is bioavailable and effectively taken up during early development. In adult zebrafish, copper accumulation was higher in the liver than in the brain, indicating a tissue-dependent distribution of Cu following Cu(OH)_2_ NP exposure.

The preferential accumulation of copper in the liver is consistent with its central role in metal homeostasis, detoxification, and metabolic regulation. Previous studies have shown that exogenous copper can undergo Cu(II) to Cu(I) redox transformation in the intestinal environment, leading to Cu(I) overflow and subsequent hepatic accumulation (Gui and Wang 2025; Yang et al. 2024; Zhang et al. 2023; Zhao et al. 2020a, b). This mechanism provides a plausible explanation for the elevated liver copper levels observed in the present study and suggests that internal copper burden, rather than nominal exposure concentration alone, is a critical determinant of toxicity.

Copper accumulation in the brain exhibited a slight increasing trend across exposure groups (not statistically significant compared to the control), suggesting that Cu(OH)_2_ NP–derived copper can reach neural tissue. Such accumulation is biologically relevant, as copper imbalance in the brain has been linked to altered neurotransmission and neurotoxicity. A previous study has demonstrated that Cu(OH)_2_ NP exposure perturbs multiple neurotransmitter pathways in developing zebrafish, supporting the notion that brain copper accumulation contributes to downstream molecular and metabolic disturbances (Chen et al. 2023).

### Alterations in copper homeostasis–related gene expression

**Fig. 2:**
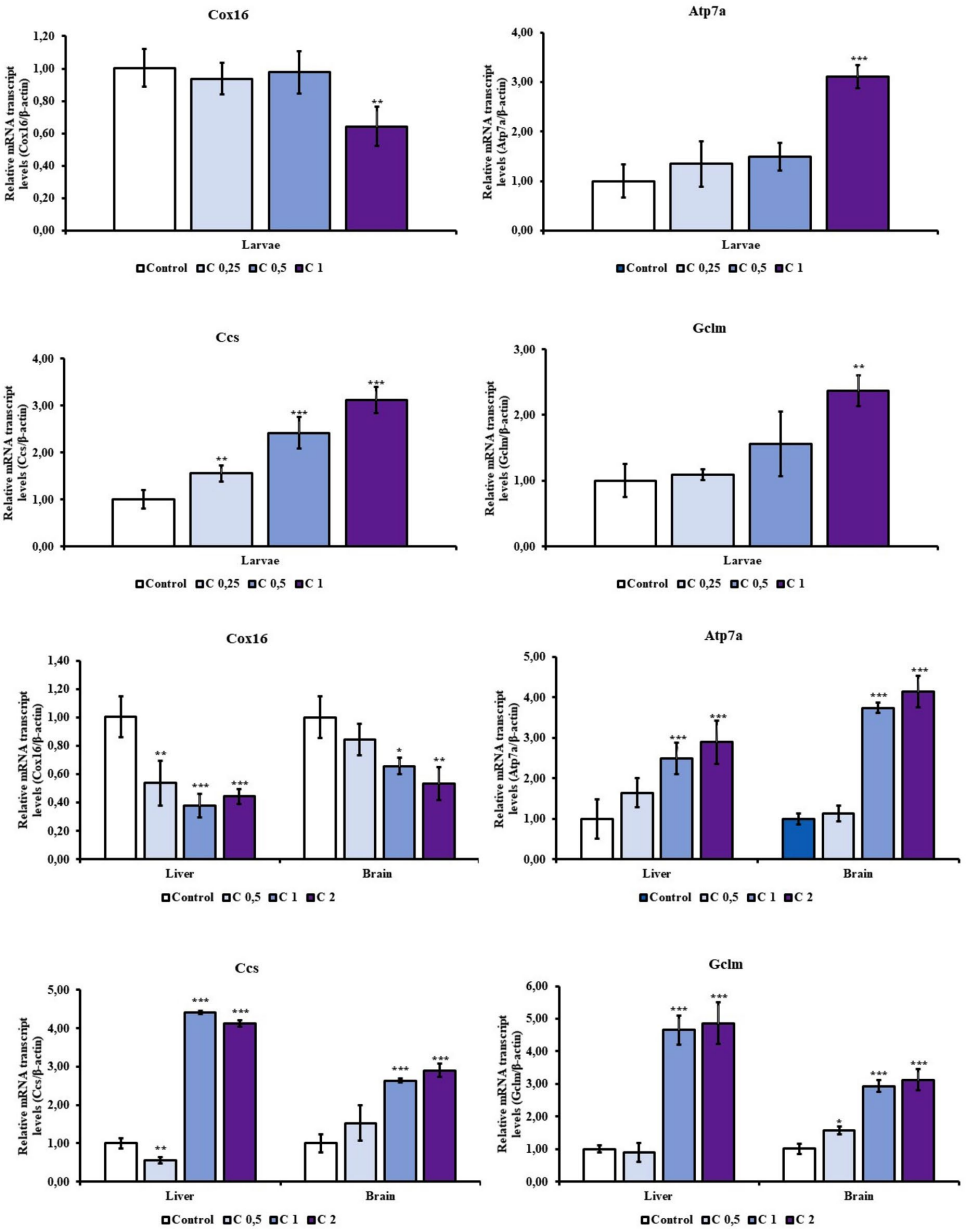
Effects of Cu(OH)_2_ NP exposure on the expression of copper homeostasis–related gene in zebrafish larvae (**A**, **B**) as well as liver and brain tissues (**C**, **D**). Gene expression levels were analyzed following Cu(OH)_2_ NP treatment, and asterisks indicate statistically significant differences compared with the control group (*P < 0.05, **P < 0.01, ***P < 0.001)

Cu(OH)₂ nanopesticide exposure significantly altered the expression of genes involved in copper transport, intracellular distribution, and redox regulation in both larvae and adult tissues (Fig. 2). In larvae, the expression of *cox16*, a gene involved in cytochrome c oxidase assembly and mitochondrial respiration, was significantly downregulated at higher exposure concentrations. In contrast, *atp7a*, encoding a copper-exporting P-type ATPase, was markedly upregulated, suggesting activation of cellular mechanisms aimed at mitigating intracellular copper overload. Similarly, *ccs*, the copper chaperone responsible for delivering copper to superoxide dismutase 1, exhibited robust induction, reflecting increased oxidative pressure and elevated demand for copper-dependent antioxidant defense. The upregulation of *gclm*, the modifier subunit of glutamate–cysteine ligase, further supports activation of glutathione biosynthesis in response to copper-induced redox imbalance (Fig. 2A and B).

In adult zebrafish, these transcriptional responses were tissue-specific. The liver displayed strong induction of *atp7a*, *ccs*, and *gclm*, consistent with its role as the primary organ for copper storage and detoxification. In addition, the brain exhibited heightened sensitivity to *cox16* downregulation, underscoring the vulnerability of neuronal bioenergetics to copper-induced stress (Fig. 2C and D). Collectively, these findings indicate that Cu(OH)₂ nanopesticide exposure perturbs intracellular copper handling and redox balance rather than merely increasing total copper content.

### Alterations in endoplasmic reticulum stress–related gene expression

**Fig. 3:**
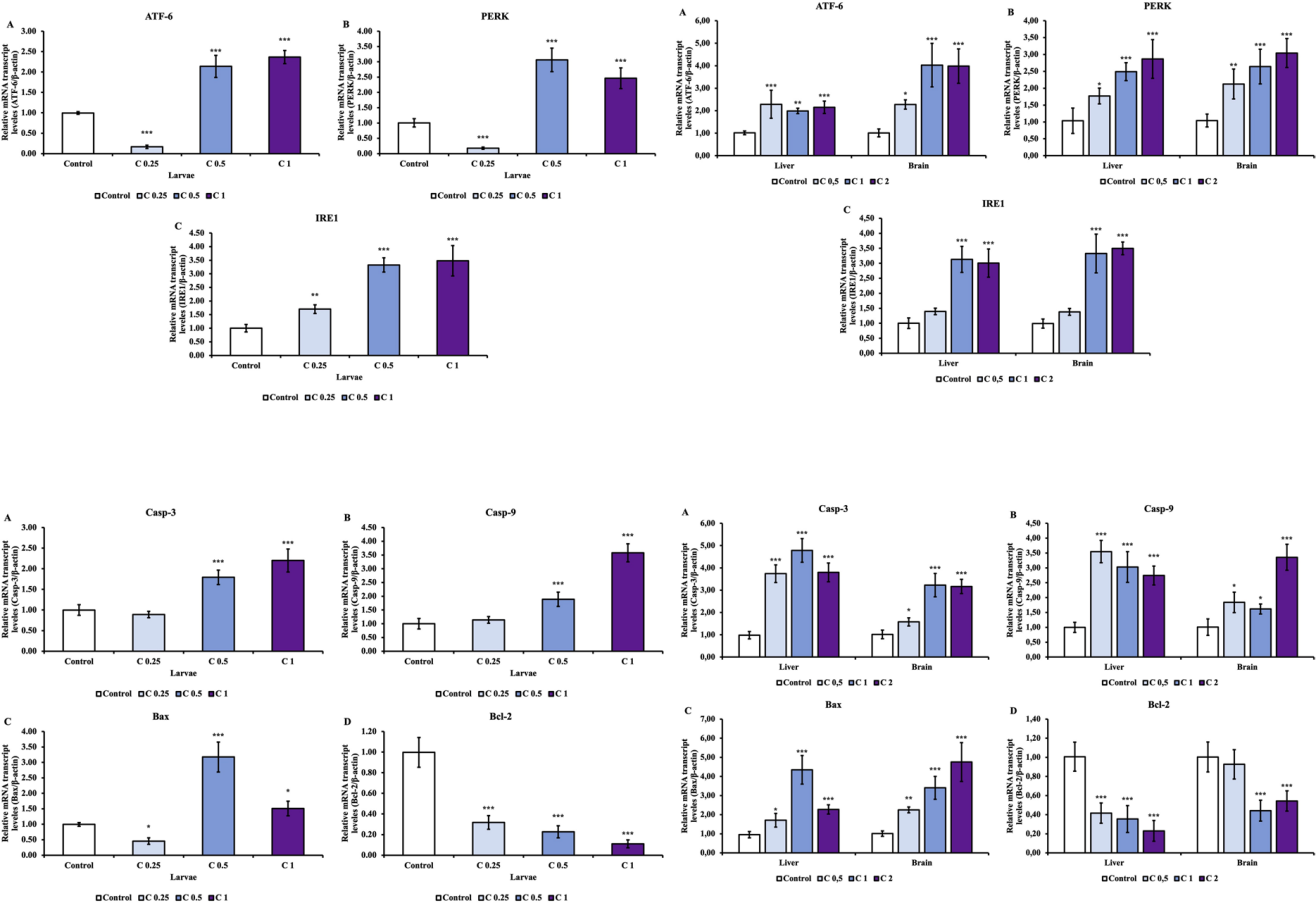
Effects of Cu(OH)_2_ NP exposure on the expression of endoplasmic reticulum (ER) stress– and apoptosis–related genes in zebrafish larvae (**A**, **B**) as well as liver and brain tissues (**C**, **D**). Gene expression levels were analyzed following Cu(OH)_2_ NP treatment, and asterisks indicate statistically significant differences compared with the control group (*P < 0.05, **P < 0.01, ***P < 0.001)

Cu(OH)_2_ NP exposure induced significant activation of endoplasmic reticulum (ER) stress signaling, as indicated by the upregulation of *atf6*, *perk*, and *ire1* in both zebrafish larvae and adult tissues (Fig. 3).

In larvae, ER stress–related genes exhibited a dose-dependent response pattern. Specifically, *atf6* and *perk* were markedly induced at moderate and high exposure concentrations, while *ire1* expression increased progressively across exposure groups (Fig. 3A–C), indicating early activation of the unfolded protein response (UPR) during development. In adult zebrafish, significant upregulation of *atf6*, *perk*, and *ire1* was observed in both liver and brain tissues (Fig. 3D–F), suggesting that Cu(OH)_2_ NP exposure elicits ER stress responses in multiple metabolically active organs rather than being confined to a single target tissue. Similar ER stress activation patterns have been reported in zebrafish and other fish species exposed to coper and copper-based nanomaterials, supporting a close link between copper dyshomeostasis and ER stress–mediated cellular responses (Liu et al. 2023; Ma et al. 2023; Zhao et al. 2020a, b).

Copper-induced redox imbalance is known to disrupt protein folding and intracellular homeostasis, rendering the ER particularly susceptible to copper-related stress. In this study, the simultaneous upregulation of *atf6*, *perk*, and *ire1* suggests engagement of a sustained UPR, which may reflect an adaptive response to prolonged cellular stress. Previous studies have demonstrated that copper accumulation and Cu(I)/Cu(II) redox cycling can impair ER function by promoting protein misfolding and oxidative modifications, particularly in metabolically active tissues such as the liver and brain (Li et al. 2024; Zhao et al. 2020a, b). Taken together, the observed ER stress responses are consistent with a cellular reaction to disrupted copper homeostasis induced by Cu(OH)_2_ NP nanopesticide exposure.

### Alterations in apoptosis-related gene expression

**Fig. 4:**
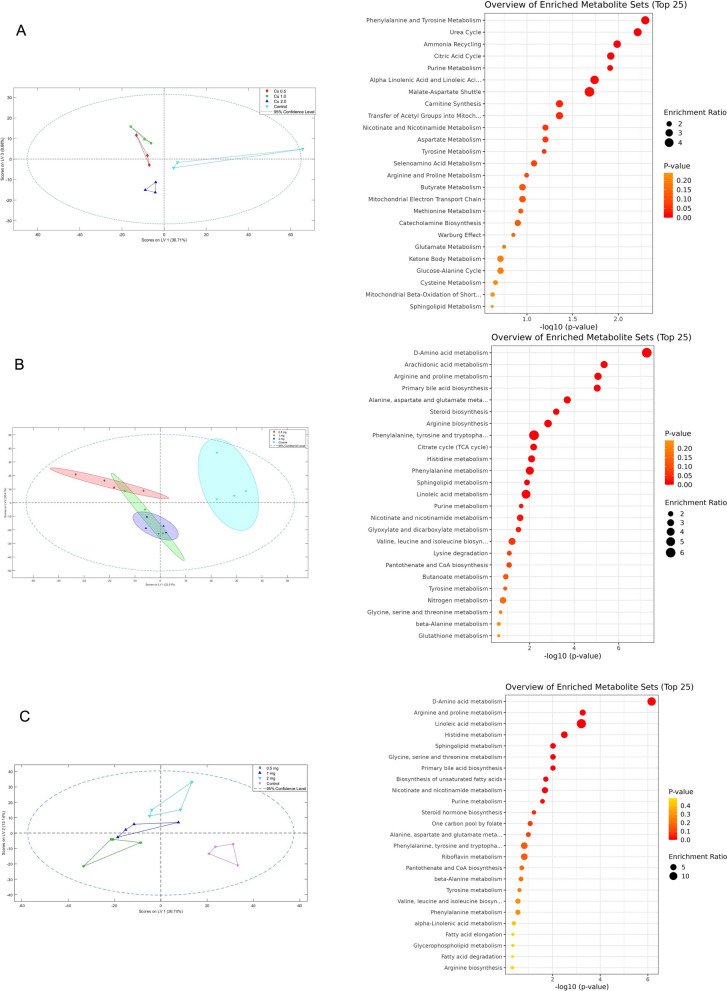
Metabolomic alterations induced by Cu(OH)_2_ NP exposure in zebrafish larvae (**A**), brain (**B**), and liver (**C**) tissues as determined by TOF–MS analysis. Orthogonal partial least squares–discriminant analysis (OPLS-DA) score plots (**A** larvae, **B** brain, **C** liver) demonstrate clear separations between control and tetraconazole-exposed groups, indicating distinct metabolomic profiles. Corresponding pathway enrichment analyses show the top 25 most significantly enriched metabolic pathways in response to Cu(OH)_2_ NP exposure, ranked according to enrichment degree and statistical significance

Consistent with the activation of endoplasmic reticulum stress, Cu(OH)₂ nanopesticide exposure significantly altered the expression of apoptosis-related genes in both zebrafish larvae and adult tissues (Fig. 4).

In larvae, transcriptional responses revealed a clear shift toward pro-apoptotic signaling. Expression of *caspase-3* and *caspase-9* was significantly upregulated at moderate and high exposure concentrations, while the pro-apoptotic gene *bax* showed marked induction, particularly at intermediate doses. In parallel, expression of the anti-apoptotic gene *bcl2* was significantly downregulated in a dose-dependent manner (Fig. 4–D). Together, these changes indicate activation of the intrinsic apoptotic pathway during early development following Cu(OH)₂ nanopesticide exposure.

In adult zebrafish, a similar pro-apoptotic transcriptional pattern was observed in both liver and brain tissues. Significant upregulation of *caspase-3*, *caspase-9*, and *bax*, accompanied by suppression of *bcl2*, suggests enhanced susceptibility to apoptosis in metabolically active organs (Fig. 4E–H). The comparable response patterns observed in liver and brain indicate that apoptotic signaling is not restricted to a single target tissue but represents a broader cellular response to sustained copper exposure.

Prolonged ER stress is known to promote apoptosis when adaptive mechanisms fail to restore cellular homeostasis. In this context, *perk* and *ire1*-dependent signaling pathways can interact with mitochondrial apoptotic machinery, leading to caspase activation. The coordinated induction of ER stress markers and apoptosis-related genes observed in the present study supports a mechanistic link between copper-induced ER stress and activation of intrinsic apoptotic pathways. Similar ER stress–associated apoptotic responses have been reported in zebrafish exposed to Cu(OH)₂ nanopesticides and other copper-based nanomaterials, suggesting that apoptosis represents a secondary response to sustained cellular stress rather than an immediate cytotoxic effect (Li et al. 2024; Zhao et al. 2020a, b).

### Metabolomic alterations associated with Cu(OH)_2_ NP exposure

Untargeted metabolomic analysis revealed exposure-related alterations in metabolic profiles following Cu(OH)_2_ NP treatment in zebrafish larvae as well as adult liver and brain tissues (Fig. 4). Multivariate analyses indicated dose-associated shifts in metabolite distributions, with exposed groups showing directional movement away from controls rather than complete separation, suggesting gradual metabolic modulation in response to increasing copper burden.

Pathway enrichment analysis identified significant perturbations in multiple interconnected metabolic pathways, including amino acid metabolism, central carbon metabolism, redox-related processes, and lipid metabolism. In larvae, enriched pathways were primarily associated with amino acid turnover (e.g., phenylalanine, tyrosine, arginine, and proline metabolism), nitrogen handling, and energy-related processes such as the tricarboxylic acid (TCA) cycle and malate–aspartate shuttle. These changes indicate early metabolic adjustments that may support developmental energy demands under copper-induced stress. Alterations in energy-related and amino acid metabolic pathways indicate that zebrafish undergo metabolic adjustments in response to copper-induced stress. Similar metabolic shifts have been reported in previous studies, where Cu(OH)_2_ NP exposure led to impaired energy metabolism accompanied by increased reliance on amino acid–based pathways (Chen et al. 2023; Wang et al. 2021).

In adult zebrafish, liver metabolomic profiles showed pronounced enrichment of pathways related to amino acid metabolism, bile acid biosynthesis, lipid metabolism, and energy production, consistent with increased metabolic and detoxification demands. Alterations in pathways linked to arginine and proline metabolism, glutamate-related processes, and mitochondrial energy pathways suggest that hepatic metabolism is reorganized to cope with sustained copper exposure. In parallel, brain metabolomic profiles exhibited enrichment of pathways associated with amino acid metabolism, sphingolipid and glycerophospholipid metabolism, and redox-related processes, indicating subtle but coordinated metabolic responses in neural tissue. Consistent with our results, previous studies have shown that Cu(OH)_2_ NP exposure disrupts multiple neurotransmitter pathways in developing zebrafish, supporting the idea that metabolic reprogramming in neural tissue may contribute to neurotoxic outcomes (Chen et al. 2023; Wang et al. 2021).

Notably, enrichment of glutathione metabolism–related pathways across tissues, together with transcriptional induction of *gclm*, points to increased antioxidant demand under copper-induced redox stress rather than depletion of cellular redox capacity. Disruptions in glutamate- and glutamine-related pathways further suggest a close interplay between energy metabolism, nitrogen balance, and redox regulation, which is particularly relevant for metabolically active tissues such as the liver and brain.

Overall, integration of metabolomic and gene expression data indicates that Cu(OH)_2_ NP exposure induces coordinated metabolic reprogramming rather than isolated pathway disruption. These metabolic changes appear to reflect an adaptive response to disrupted copper homeostasis and increased cellular stress, which may become maladaptive under sustained or higher exposure conditions. Such systems-level metabolic adjustments are consistent with the observed activation of ER stress signaling and apoptosis-related pathways, supporting a unified mechanistic framework for Cu(OH)_2_ NP–induced toxicity.

### Effects of Cu(OH)_2_ NP exposure on adult zebrafish health indices

The organism-level toxic effects of Cu(OH)_2_ NP exposure were evaluated using biometric indices, including condition factor (CF), body mass index (BMI), and hepatosomatic index (HSI) (Table 2). Neither CF nor BMI values showed significant differences among exposure groups after the 14-day exposure period, indicating that overall somatic condition and body mass–related growth were largely preserved under the tested exposure concentrations. In contrast, HSI exhibited a marked and dose-dependent increase in Cu(OH)_2_-exposed adult zebrafish, with a statistically significant elevation observed in the highest exposure group (2.0 mg L⁻^1^) compared to the control.

**Table 2:** Impacts of Cu(OH)_2_ on condition factor (CF), body mass index (BMI), and hepatosomatic index (HSI) of adult zebrafish after exposure to the fungicide for 14 days

Groups	CF	BMI	HSI
Control	1.16 ± 0.04^a^	0.036 ± 0.009^a^	1.373 ± 0.205^a^
0.5 mg/L	0.97 ± 0.09^a^	0.030 ± 0.002^a^	1.430 ± 0.350^a^
1.0 mg/L	1.14 ± 0.04^a^	0.036 ± 0.007^a^	1.658 ± 0.653^a^
2.0 mg/L	1.18 ± 0.02^a^	0.035 ± 0.006^a^	2.330 ± 0.233^*b^

Data are presented as mean ± SD (*n* = 15). According to the post hoc Duncan test, the difference between the groups with different letter in the same column is significant (*p* < 0.05). According to the one-way Anova Dunnett, the difference between the groups with asterisks in the same column and the control group is significant (*p* < 0.05)

HSI is widely recognized as a sensitive biomarker of liver health status and reflects hepatic enlargement and functional alteration under xenobiotic stress (Figueiredo-Fernandes et al. 2007; Narra 2016; Sánchez et al. 2017). Previous studies have demonstrated that increased HSI is an important toxicological indicator associated with exogenous chemical exposure, often linked to hepatocellular hypertrophy, hyperplasia, or lipid accumulation (Figueiredo-Fernandes et al. 2007; Weinhouse et al. 2014). Consistent increases in HSI have been reported in adult zebrafish exposed to various pesticides and environmental contaminants, including Cu and bisphenol analogues, supporting the sensitivity of this index in detecting liver-specific toxicity (Pan et al. 2019; Weinhouse et al. 2014). In the present study, the selective elevation of HSI, in the absence of significant changes in CF and BMI, suggests that Cu(OH)_2_ NP exposure primarily targets hepatic function rather than inducing generalized growth impairment.

Importantly, the increased HSI observed in adult zebrafish aligns closely with the molecular and metabolomic alterations detected in liver tissue. The induction of endoplasmic reticulum stress–related genes and apoptosis-associated markers, together with metabolomic evidence of disrupted energy and amino acid metabolism, indicates that hepatic enlargement may represent an adaptive response to increased metabolic and detoxification demands imposed by copper accumulation. Given the central role of the liver in copper homeostasis, detoxification, and metabolic regulation, sustained disruption of copper balance is likely to impose a substantial hepatic burden, ultimately leading to altered liver function and morphology.

In the present study, the toxicity of Cu(OH)_2_ NP was assessed through an integrated analysis at the end of a 14-day exposure period. This single-point terminal analysis revealed significant alterations in the hepatosomatic index (HSI), internal copper accumulation, and systemic metabolic profiles. The marked increase in HSI in the highest exposure group suggests that 14 days is a sufficient duration to induce observable physiological and morphological changes in the liver, consistent with previous reports on hepatic stress in zebrafish (Pan et al. 2019; Zulfahmi et al. 2025). Furthermore, our metabolomic data demonstrated broad-spectrum reprogramming of energy and amino acid pathways. Such profound shifts in the metabolome reflect a consolidated biological response to sustained copper stress rather than transient early-stage fluctuations (Chen et al. 2023; Lu et al. 2024; Wang et al. 2024a, b). Therefore, the molecular and metabolic signatures observed on Day 14 provide a robust basis for concluding the cumulative toxicity of Cu(OH)_2_ NP, representing the transition from adaptive homeostatic mechanisms (e.g., upregulation of *atp7a*) to maladaptive cellular outcomes such as ER stress and apoptosis.

## Conclusions

In this study, the effects of exposure to Cu(OH)_2_ NP were evaluated in zebrafish larvae and adults using a combination of molecular, metabolomic, and organism-level endpoints. Exposure to Cu(OH)_2_ NP resulted in increased internal copper levels, with higher accumulation observed in adult liver tissue, indicating effective uptake and tissue-specific distribution of nanopesticide-derived copper.

Alterations in genes associated with copper handling, cellular stress responses, and apoptosis suggest that disruption of copper homeostasis occurs following Cu(OH)_2_ NP exposure. Metabolomic analyses further revealed changes in energy-related and amino acid metabolic pathways, indicating metabolic adjustments under increased copper burden. At the organism level, the observed increase in hepatosomatic index, in the absence of marked changes in general growth parameters, points to the liver as a sensitive target organ.

Overall, the findings indicate that Cu(OH)_2_ NP exposure is associated with coordinated molecular and metabolic changes in zebrafish rather than isolated toxic effects. These results contribute to a better understanding of the biological responses to copper-based nanopesticides and may support future evaluations of their environmental safety.

## Data Availability

No datasets were generated or analyzed during the current study.
